# Complete Genome Sequence of a Vaccinal Newcastle Disease Virus Strain Isolated from an Owl (*Rhinoptynx clamator*)

**DOI:** 10.1128/genomeA.01243-16

**Published:** 2016-11-17

**Authors:** Steven Van Borm, Laís S. Rizotto, Leila S. Ullmann, Guilherme P. Scagion, Camila D. Malossi, Raphael M. Simão, João P. Araújo, Izabelle M. Cordeiro, Lara B. Keid, Trícia Maria F. Sousa Oliveira, Rodrigo M. Soares, Matheus C. Martini, Maria A. Orsi, Clarice W. Arns, Helena L. Ferreira

**Affiliations:** a Department of Veterinary Medicine, Faculty of Animal Science and Food Engineering, University of São Paulo (FZEA-USP), Pirassununga, Brazil; b Postgraduate Program in Experimental Epidemiology of Zoonoses, Faculty of Veterinary Medicine and Animal Science, University of São Paulo (FMVZ-USP), São Paulo, Brazil; c Molecular Platform Unit, CODA-CERVA Veterinary and Agrochemical Research Centre, Brussels, Belgium; d Laboratory of Animal and Human Virology, Department of Microbiology and Immunology, Biosciences Institute, Universidade Estadual Paulista, Botucatu, UNESP, São Paulo, Brazil; e Laboratory of Animal Virology, Institute of Biology, University of Campinas (UNICAMP), Campinas, Brazil; f National Agricultural Laboratory of Brazil, Lanagro/São Paulo, Campinas, Brazil

## Abstract

A Newcastle disease virus (NDV) was isolated in chicken embryonated eggs after detection by real-time reverse transcription-PCR (RRT-PCR) from a captive owl swab. The complete genome sequence of APMV-1/*Rhinoptynx clamator*/Brazil/22516/2009 (APMV-1, avian paramyxovirus type 1) was obtained using Illumina sequencing. Phylogenetic analysis of the complete genome classified the isolate within NDV class II genotype II.

## GENOME ANNOUNCEMENT

In Brazil, Newcastle disease virus (NDV), an avian paramyxovirus type 1 (APMV-1), is an economically important poultry disease that can be maintained in wild birds (1–3).

During an ongoing wild bird surveillance project in the Southeast of Brazil, São Paulo state, an NDV was isolated after three passages in embryonated chicken eggs, as previously described (4), from a real-time RT-PCR-positive (as previously described [5]) oropharyngeal swab taken from a clinically healthy striped owl (*Rhinoptynx clamator*).

The virus isolate (allantoic fluid) was centrifuged at 8,000 rpm for 2 min at 4°C and filtered with 0.45-µm disk filters (Millipore), followed by a nuclease treatment (50 U of Ambion Turbo DNase in 150 µl with incubation at 37°C for 1 h) and RNA purification using TRIzol LS (Invitrogen) with purification of the aqueous phase containing the RNA using the QIAamp viral RNA minikit (Qiagen). The RNA concentration was quantified using a DS-11 spectrophotometer (DeNovix) and a Qubit fluorometer (Invitrogen). cDNA was synthesized using the SuperScript IV first-strand synthesis system (Invitrogen) and random hexamer primers, followed by double-strand cDNA synthesis using T4 DNA polymerase and T4 DNA ligase (Thermo Scientific), as previously described (6). Nextera XT libraries (Illumina) were prepared using 1 ng of double-stranded cDNA (6), quantified with a Kapa library quantitation kit Illumina Platforms (Kapa Biosystems) diluted to 1 nM, and sequenced on the NextSeq system (Illumina, Inc., San Diego, CA, USA) using a NextSeq 500 mid output kit (1 × 150 cycles) (Illumina Inc.). The quality of the sequences was checked with FastQC version 0.10.1 (http://www.bioinformatics.babraham.ac.uk/projects/fastqc/). Stretches containing unidentified nucleotides (“N”) were trimmed using Cutadapt version 1.3 (7) prior to quality trimming using Sickle version 1.210 (*Q* score, <30; length, <50 bp) (8). Host reads were removed by mapping to the *Gallus gallus* genome (accession no. GCF_000002315.4) (bwa version 0.7.12 [9]). Fifty thousand randomly selected unmapped reads were used for a *de novo* assembly (Newbler version 2.9; Roche), resulting in a 15,110-bp NDV contig. All *G. gallus* unmapped reads (2,053,959 reads) were subsequently mapped (Newbler version 2.9; Roche) to a hybrid reference consisting of the 15,110-bp *de novo* contig supplemented with the missing 5′ noncoding region (76 nucleotides) from its closest BLASTn hit, accession no. KM056358 (4).

The complete genome sequence of NDV isolate APMV-1/*Rhinoptynx clamator*/Brazil/22516/2009 was thus assembled (average coverage, 1,971×) as a 15,186-bp contiguous sequence, confirming to the rule of six described for paramyxoviruses (10). The protein-coding genes were predicted relative to reference sequence KM056358 by GATU (11)

Phylogenetic analysis of the complete genome classified the isolate as members of NDV class II, genotype II (data not shown). Pathogens can be transmitted from domestic animals to free-ranging hosts and vice versa (12, 13). Indeed, the continuous expansion of the poultry industry coupled with the mass employment of live-virus vaccines (14) may result in a spillover of vaccinal strains to wildlife reservoirs (15). The impact of these vaccinal strains in wildlife reservoirs remains unknown.

### Accession number(s).

The complete genome sequence of NDV isolate APMV-1/*Rhinoptynx clamator*/Brazil/22516/2009 has been deposited in GenBank under accession number KX822746.
